# On Medical Theories

**Published:** 1807-01

**Authors:** 


### To the Editors of the Medical and Physical Journal.

GENTLEMEN,

AS medical theories are valuable only in proportion as they lead to successful practice in the cure of diseases, I cannot help thinking that more benefit is likely to result to the healing art from the collected observations of experienced practitioners, who, unbiassed by system, accurately discriminate and faithfully record, than from any further attempts to generalise, however transcendent in ingenuity. I am gratified by perceiving that a disposition to empirical observation seems to be taking place of that rage for systematising which characterises most of our modern productions, and I regard with a degree of veneration the patient investigating spirit of a Heberden and a Haygarth. I am not an enemy to theory, but I see much greater reason to apprehend that *its* influence may tend to the distortion and suppression of truth, than that any attention to facts can ever have the effects of paralysing our reasoning faculties. To think and to reason are with me convertible terms; every thinking man is a theorist, and the distinction between dogmatists and empirics in physic has long since been proved to consist in degree only.

With these impressions I feel myself justified in calling on every member of the profession to register his daily observations; to mark the features of disease, discriminating such symptoms as sanction the prevailing theory from such as are repugnant thereto; to note the efficacy and inefficacy of the remedies employed, with the circumstances under which they fail or succeed; and, finally, to record publicly the result of such observations as soon as they become sufficiently numerous and marked by sufficient uniformity to warrant their receiving the public confidence. This simple mode of investigation I conceive preferable to the framing of precipitate analogies; preferable also to the recording individual histories of diseases, be their terminations ever so extraordinary; it is less liable to error, and its results may be applied with safety and effect in immediate practice. Independant of all theory it connects not itself with any classification of diseases, with any one of which our enquiries may of course commence.

From certain considerations pressing on my own mind I could wish the first clinical deductions which may appear, to be on the subject of hydrocephalus internus, a disease which which affords a striking illustration how much such investigation as I have been urging is called for. No disease has excited greater or more justly founded alarm, yet is its clinical history still obscure; and much as Dr. Quin, and several subsequent writers, have contributed to elucidate its nature, an ample field yet remains for observation and research.

To the essay of Quin I attribute a high degree of merit; but I much fear that his reasonings have universal application, and that practitioners in the satisfaction they experienced from arriving at some fixed principles in the contemplation of a disease so long involved in utter darkness, have been lulled into quiescence, and in regarding the more acute disease of Quin have overlooked a still more insidious and formidable one, unmarked by any acute symptoms. The existence of an inflammatory stage succeeded by a well-known train of contrary symptoms having been once clearly proved, it was a natural error for those who considered the latter as resulting from the former, to conceive that the former *always* preceded the latter. I have some reason for believing the contrary; I have reason to think that an affection of the brain occurs frequently in children, and even much more frequently than the acute disease of Quin, in which neither from the symptoms nor dissections can any inflammatory action in the vessels of the brain be discovered.

In reviewing many of the cases recorded as of hydrocephalus internus, I find the proofs of inflammatory origin completely equivocal; in short, I see much reason for concurring in the sentiments of an ingenious medical friend, which is, that there are two species of the disease, a chronic as well as an acute, including in this distinction only the apoplexia hydrocephalica of Cullen, without any reference to the dropsical hydrocephalus of that author, altho' future investigation may possibly trace a closer connexion between these than has heretofore been supposed to exist.

To establish such a distinction, if founded in truth, and to adapt to each species its appropriate treatment, should be the object of the clinical observer; on this subject therefore I would wish now to elicit, through the medium of your Journal, and from such practitioners as have had sufficient opportunity for forming their experience, succinct reports of such cases as have occurred to them, stating the age, sex, constitution, mode of early sustenance, *whether by breast or spoon,* premonitory symptoms, symptoms of attack, duration antecedent to medical treatment, continuance after, event, and above all, where this has been unfortunate, the appearances on dissection. Such representations I consider as most conveniently given in tabular form, attaching all illustrations and deductions in form of appendix.

In this way, both the time and patience of readers are less trespassed on than where cases are more minutely and individually detailed, at the same time that the similitudes and discrepancies are rendered more obvious and impressive than if the leading features are committed to memory and afterwards compared, a necessity inseparable from the practice of giving minute histories of disease in successive detail.

By this mode too the facts are presented to view, distinct from the theoretic opinions of the observer, a circumstance of which the value will be estimated by all such as have experienced the seductive influence which the ingenious speculations of an author possess over the mind of his reader, however dispassionate, who too often sees the facts only through the medium of the relater, and pleased with the progress of the visionary fabric erecting before him, stops not to enquire into the permanence of its foundation, but applying to scientific discussion the maxim of a celebrated author, the application of which was never intended to extend beyond works of imagination, he gives up the reins of his own into his author's hands, is pleased, knows not why, and cares not wherefore.

I now conclude, in the hope that in embodying the preceding reflections I have not beeu unprofitably employed.

